# *Kochia scoparia* Saponin Momordin Ic Modulates HaCaT Cell Proliferation and Apoptosis via the Wnt/*β*-Catenin Pathway

**DOI:** 10.1155/2021/5522164

**Published:** 2021-07-16

**Authors:** Meijunzi Luo, Bijun Zeng, Haizhen Wang, Zhibo Yang, Youhua Peng, Yujin Zhang, Chang Wang

**Affiliations:** Department of Dermatology, The Second Affiliated Hospital, The Domestic First-Class Discipline Construction Project of Chinese Medicine of Hunan University of Chinese Medicine, Changsha, Hunan 410005, China

## Abstract

Psoriasis is a chronic, recurrent, immunoinflammatory disease. For a long period, Traditional Chinese Medicine (TCM) is considered a reliable alternative therapy for patients with psoriasis. Fructus Kochiae (or *Kochia scoparia*) and its principle saponin, Momordin Ic, have been reported to protect against inflammation. Herein, we demonstrated that Momordin Ic could inhibit HaCaT cell proliferation and enhance cell apoptosis. In the meantime, Momordin Ic alters Wnt/*β*-catenin pathway activation by affecting *β*-catenin nuclear distribution. The Wnt/*β*-catenin signaling activator LiCl partially reversed the effects of Momordin Ic on HaCaT phenotypes and the Wnt/*β*-catenin pathway factors. Altogether, we demonstrate the inhibitory effects of Momordin Ic, one of the major saponin constituents of Fructus Kochiae, on HaCaT cell proliferation and Momordin Ic-induced alteration within the Wnt/*β*-catenin pathway. Momordin Ic might act on HaCaT cells by modulating the Wnt/*β*-catenin pathway.

## 1. Introduction

Psoriasis is a chronic, recurrent, immunoinflammatory disease. The most common syndromes in patients consist of pain, itching, and bleeding. The burden of disease continues to be enhanced by a number of comorbidities, including metabolic syndromes and cardiovascular disorders caused by the syndromes [1, 2]. Moreover, visible disfigurement could cause negative reactions from others, resulting in many easily measured psychological burdens [3, 4]. Psoriatic pathogenesis is complicated, which can be related to heredity, immune system, environment; thus, there is no effective cure for psoriasis by far [5]. Sterols commonly used for psoriasis treatment and systemic therapy based on monoclonal antibodies and inhibitors are often accompanied by side effects such as gastrointestinal intolerance and elevated blood lipid [6]. Therefore, it is of great importance to find effective alternative therapies for psoriasis.

Traditional Chinese Medicine (TCM) is characterized by multiple pathways, target points, and elements, and its complex composition can further enhance the acting surface and effect intensity of the drug target. For a long time, TCM has been considered a reliable alternative therapy for patients with psoriasis. A variety of prescriptions for psoriatic treatment were documented in ancient Chinese medical books, such as The Peaceful Holy Benevolence Formulae and Taiping Holy Prescriptions for Universal Relief [7]. Fructus Kochiae is the fruit of *Kochia scoparia* (L.) Schrad., a broom cypress fruit, which has been used as an edible and topical drug in the treatment of skin, urinary, and eye diseases for more than 2000 years in China [8, 9]. Fructus Kochiae (or *Kochia scoparia*) has been reported to protect against skin inflammation related to evil heat, humidity, and wind, including atopic dermatitis [10–14]. Reportedly, one of the major saponin constituents of Fructus Kochiae, Momordin Ic, has a number of biological activities, including controlling glucose-induced elevated blood sugar levels [8], inhibiting gastric emptying [15], antirheumatoid arthritis activity [15], and reducing hepatotoxicity caused by carbon tetrachloride [16]. However, the specific functions of Momordin Ic on psoriasis remain unclear.

Notably, Momordin Ic has been reported to be associated with the apoptosis (proliferation) of multiple cell types, including hepatocellular carcinoma HepG2 cells [17–19] and prostate cancer cells [20]. Our previous study also indicated Momordin Ic inhibition upon the proliferation of HaCaT cells. According to previous studies, psoriasis is mainly characterized by excessive epidermal proliferation and keratinocyte premature maturation followed by partial cornification and retention of nuclei within the stratum corneum [21]. The imbalance between keratinocyte proliferation and differentiation leads to hyperproliferative epidermis beyond the extent of skin damage [22]. Thus, inhibiting the hyperproliferation of keratinocytes has long been considered a potential strategy for psoriasis treatment [23–25]. The Wnt/*β*-catenin pathway exerts an important effect on activating and maintaining the proliferation of keratinocytes during hair follicle cycling [26]. In promoting the proliferation of basal keratinocytes in the interfollicular epidermis, paracrine Wnt signaling and *β*-catenin transcriptional activity are essential [27, 28]. As for psoriasis, previous studies also indicated that the Wnt/*β*-catenin pathway was involved in the proliferation of HaCaT cells under IL-22 stimulation [29] or without treatment [30]. Thus, the hyperproliferation of keratinocytes, which might be promoted by the Wnt/*β*-catenin pathway, has been reported to be a critical event that should be considered for psoriasis treatment.

Considering the above-mentioned findings, we speculate that Momordin Ic might also inhibit keratinocyte proliferation, possibly via the Wnt/*β*-catenin pathway. To validate the speculation, we first treated the human immortalized epidermal cell line, HaCaT, with increasing concentrations of Momordin Ic and chose the minimum efficient concentration. The effects of Momordin Ic on HaCaT cell viability, apoptosis, cell cycle distribution and the key factors of the Wnt/*β*-catenin pathway were examined. We also cotreated HaCaT cells with Momordin Ic and Wnt/*β*-catenin pathway activator (LiCl) for verifying that the Wnt/*β*-catenin pathway was involved. Taken together, we examined the specific effects of Momordin Ic on HaCaT cell proliferation and the Wnt/*β*-catenin pathway, attempting to provide a rationale for further studies on Momordin Ic cellular functions in HaCaT cells.

## 2. Materials and Methods

### 2.1. Cell Line and Cell Culture

Human immortalized epidermal cell line, HaCaT (3142C0001000001712), was obtained from China Center for Type Culture Collection (CCTCC; Wuhan, China) and cultured in Minimum Essential Medium (MEM Eagles with Earle's Balanced Salts; Gibco, Waltham, MA, USA) added with 10% FBS (Invitrogen, Carlsbad, CA, USA) at 37°C in 5% CO_2_.

### 2.2. Cell Treatments

Momordin Ic was purchased from Yuanye Biotech (CAS: 96990-18-0, HPLC ≥ 98% Shanghai, China). LiCl was purchased from Sigma-Aldrich (CAS: 7447-41-8, Shanghai, China). HaCaT cells were treated with different concentrations of Momordin Ic (5, 12.5, 25, 50, or 100 *μ*mol/L) or 30 mM LiCl for 24 or 48 h. The same amount of PBS was used as control. Then, cells were harvested for further experiments.

### 2.3. MTT Assay for Cell Viability

Cells were transfected and/or treated with Momordin Ic, washed with normal culture medium, and added with 10 *μ*l MTT solution (10 mg/ml) to incubate the cells for 4 h. At the end of the incubation, 100 *μ*l DMSO solution was added to incubate the cells for 10 min. Then, detect the OD value at 490 nm with a microplate reader. The lowest effective concentration of Momordin Ic was selected.

### 2.4. Flow Cytometry for Cell Apoptosis and Cell Cycle

Cells were treated with Momordin Ic for 24 h, 48 h, and 72 h, centrifuged with PBS, and added with RNaseA. Resuspend the cells in Annexin V-FITC binding solution, add Annexin V-FITC fluorescein solution and propidium iodide (PI) staining solution for cell apoptosis detection, or add PI staining solution for cell cycle detection. The experiment was repeated three times.

### 2.5. qRT-PCR

Logarithmic phase HaCaT cells were collected to extract total RNA using a TRIzol™ Plus RNA Purification Kit (Thermo Fisher Scientific, Waltham, MA, USA); then, the reverse transcription into cDNA with a High-Capacity cDNA Reverse Transcription Kit was performed (Applied Biosystems, Loughborough, UK), and the mRNA expressions of *β*-catenin, c-myc, and VEGF were detected, as shown in Table 1.

### 2.6. Immunoblotting

The total protein of HaCaT cells was extracted with a Total Protein Extraction Kit (Millipore, Burlington, MA, USA). The protein concentration was determined using a bicinchoninic acid (BCA) method with a Pierce™ BCA Protein Assay Kit (Thermo Fisher Scientific). Protein samples were separated on a 10% SDS-PAGE and then transferred to a PVDF membrane. After blocking with 5% (w/v) skimmed milk at room temperature for 1 h, protein samples were incubated with the corresponding primary antibody at 4°C overnight. The following antibodies were used: anti-*β*-catenin (51067-2-AP; Proteintech, Wuhan, China), anti-c-Myc (10828-1-AP, Proteintech), and anti-VEGF (66828-1-Ig, Proteintech). Then, protein samples were incubated with the secondary antibody. Band signals were visualized by the enhanced chemiluminescence (ECL, Bio-RAD, Hercules, CA, USA) method according to the manufacturer's instructions. Relative protein expression was normalized to actin.

### 2.7. Immunofluorescence Staining

The HaCaT cells were fixed with 4% paraformaldehyde for 20 min, washed with PBS, and permeabilized with 0.1% Triton X-100 for 10 min. Then, the cells were blocked in PBS containing 10% BSA at room temperature for 1 h. At the end of the blocking, cells were incubated with anti-*β*-catenin (51067-2-AP; Proteintech) at 4°C overnight. Then, the cells were stained with a proper FITC-conjugated secondary antibody (1 : 1000) in a dark room at 37°C for 1 h. Then, DAPI was used to stain the nucleus. The cells were observed under the inverted fluorescence microscope.

### 2.8. Data Processing and Statistical Analysis

The data were analyzed with GraphPad software. The measurement data were expressed as mean ± standard deviation (SD). Among-group and intragroup data comparisons were performed with the ANOVA followed by Tukey's post hoc test. *P* < 0.05 indicated statistically significant difference.

## 3. Results

### 3.1. Effects of Different Concentrations of Momordin Ic on HaCaT Cell Survival Rate

**Figure 1:**
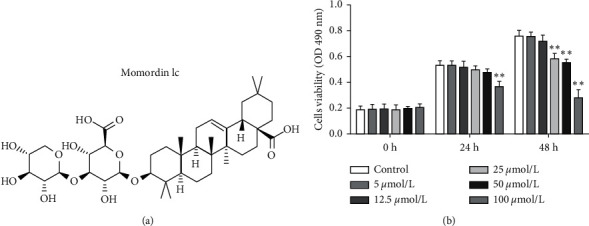
Effects of different concentrations of Momordin Ic on HaCaT cell survival rate. (a) The chemical structure of Momordin Ic. (b) HaCaT cells were treated with 5, 12.5, 25, 50, and 100 *μ*mol/L Momordin Ic for 0, 24, or 48 h; the cell viability was determined by MTT assay. ^*∗∗*^*P* < 0.01.

Before investigating the specific effects of Momordin Ic (Figure 1(a)) on HaCaT cell phenotypes, we firstly treated HaCaT cells with 5, 12.5, 25, 50, or 100 *μ*mol/L Momordin Ic for 0, 24, or 48 h and examined the cell viability. Compared with the nontreated group (control), after 48 h of treatment, 25, 50, and 100 *μ*mol/L significantly reduced the HaCaT cell viability. The IC50 of Momordin Ic is 168.70 and 76.40 *μ*mol/L for 24 and 48 h, respectively. Thus, 25 *μ*mol/L Momordin Ic treatment for 48 h was selected for further experiments.

### 3.2. Momordin Ic Causes Cell Apoptosis and Cell Cycle Arrest within HaCaT Cells

**Figure 2:**
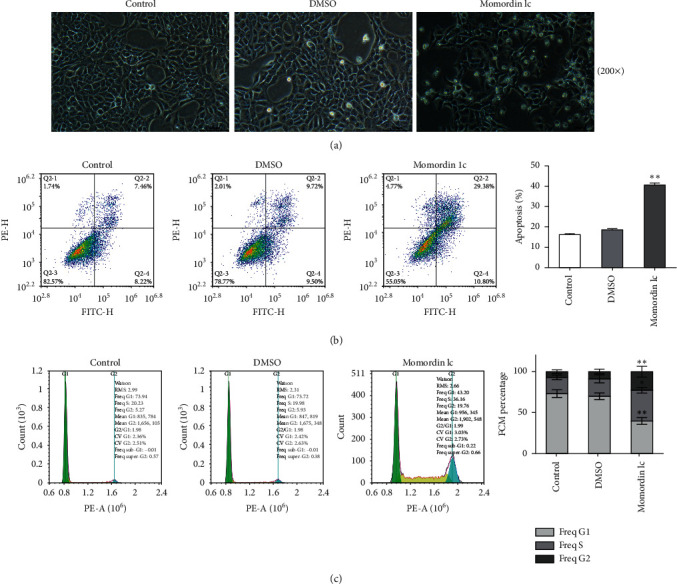
Momordin Ic induces apoptosis and cell cycle arrest in HaCaT cells. Target cells were treated with 25 *μ*mol/L Momordin Ic for 48 h and examined for the morphology changes under an inverted microscope (a) (scale bar is 100 *μ*m); cell apoptosis and cell cycle using flow cytometry (b-c). ^*∗*^*P* < 0.05, ^∗∗^*P* < 0.01.

Next, we treated HaCaT cells with 25 *μ*mol/L Momordin Ic for 48 h and examined for the morphological and phenotype changes. Compared with the blank control and DMSO treatment groups, HaCaT cells in the Momordin Ic group were shrunk, the volume was reduced, and the number of dead cells increased (Figure 2(a)). As for cellular functions, Momordin Ic treatment significantly promoted cell apoptosis (Figure 2(b)) and caused S-phase arrest of cell cycle (Figure 2(c)), compared with the blank control and DMSO treatment groups.

### 3.3. Momordin Ic Treatment Inhibits the Wnt/*β*-Catenin Pathway within HaCaT Cells

**Figure 3:**
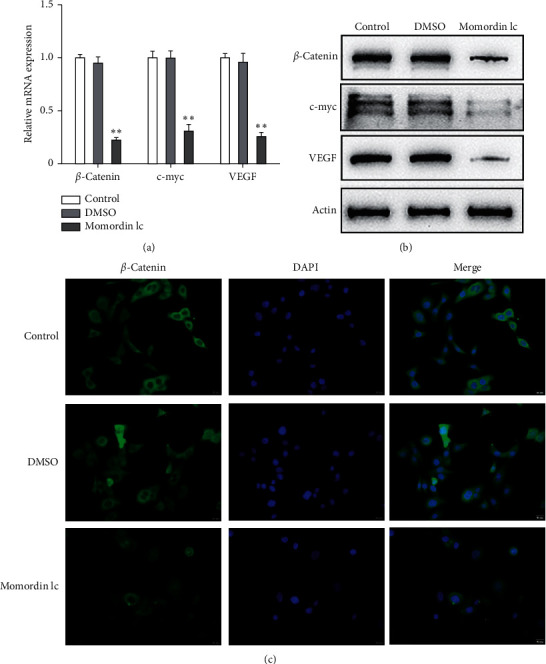
Momordin Ic treatment inhibits the Wnt/*β*-catenin signaling in HaCaT cells. Target cells were treated with 25 *μ*mol/L Momordin Ic for 48 h and examined for the mRNA expression of *β*-catenin, c-myc, and VEGF by qRT-PCR (a); the protein levels of *β*-catenin, c-myc, and VEGF by immunoblotting (b); the content and distribution of *β*-catenin by immunofluorescent (IF) staining (c) (scale bar is 20 *μ*m). ^∗∗^*P* < 0.01.

Considering the critical effect of the Wnt/*β*-catenin signaling on HaCaT cell growth, next, we investigated the potential roles of Momordin Ic treatment in the Wnt/*β*-catenin signaling. We treated target cells with 25 *μ*mol/L Momordin Ic for 48 h and examined for *β*-catenin, c-Myc, and VEGF mRNA and protein expression. As shown in Figures 3(a) and 3(b), Momordin Ic treatment significantly decreased *β*-catenin, c-Myc, and VEGF mRNA and protein expression, compared with the blank control and DMSO treatment groups. More importantly, IF staining showed that in the Momordin Ic treatment group, the localization of *β*-catenin was within the nucleus (Figure 3(c)).

### 3.4. Momordin Ic Acts on HaCaT Cells via the Wnt/*β*-Catenin Pathway

**Figure 4:**
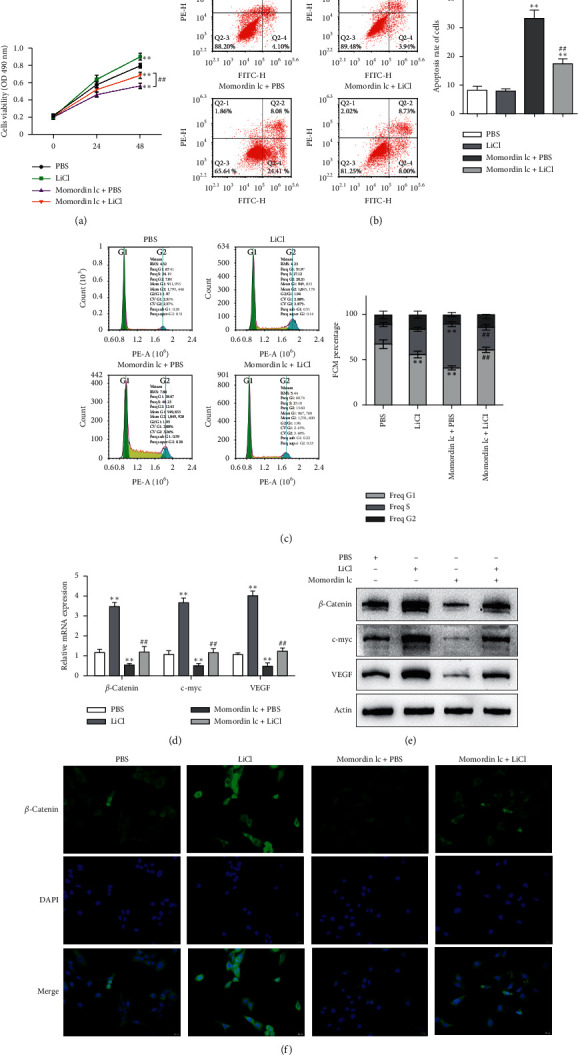
Momordin Ic acts on HaCaT cells through the Wnt/*β*-catenin signaling. Target cells were treated with PBS, 30 mM LiCl, 25 *μ*mol/L Momordin Ic + PBS, or 25 *μ*mol/L Momordin Ic + 30 mM LiCl for 48 h and examined for cell viability by MTT assay (a); cell apoptosis and cell cycle by flow cytometry (b-c); the mRNA expression of *β*-catenin, c-myc, and VEGF by qRT-PCR (d); the protein levels of *β*-catenin, c-myc, and VEGF by immunoblotting (e); the content and distribution of *β*-catenin by immunofluorescent (IF) staining (f) (scale bar is 20 *μ*m). ^*∗*^*P* < 0.05, ^∗∗^*P* < 0.01.

Since Momordin Ic treatment altered the Wnt/*β*-catenin pathway within HaCaT cells, here, we determined whether Wnt/*β*-catenin pathway activator (LiCl) could reverse Momordin Ic effects on HaCaT cells. Compared to the PBS control group, LiCl treatment increased the cell viability, while Momordin Ic reduced the cell viability and increased cell apoptosis (Figures 4(a) and 4(b)). Under cotreatment of Momordin Ic and LiCl, LiCl obviously enhanced cell viability (Figure 4(a)) and repressed Momordin Ic-induced cell apoptosis (Figure 4(b)). Moreover, Momordin Ic-induced cell S stage cycle arrest was also reversed by LiCl cotreatment (Figure 4(c)). Compared to the PBS control group, LiCl treatment increased *β*-catenin, c-Myc, and VEGF mRNA and protein expression. Momordin Ic suppressed *β*-catenin, c-Myc, and VEGF mRNA and protein expression, which were partially reversed via LiCl cotreatment (Figures 4(d) and 4(e)). Furthermore, IF staining showed that LiCl increased the cellular fluorescent signaling of *β*-catenin. Momordin Ic reduced the fluorescence of *β*-catenin, which was partially reversed by LiCl cotreatment (Figure 4(f)).

## 4. Discussion

Herein, the study revealed that one of the major saponin constituents of Fructus Kochiae, Momordin Ic, could inhibit HaCaT cell proliferation and enhance cell apoptosis. In the meantime, Momordin Ic also alters Wnt/*β*-catenin pathway activation. The Wnt/*β*-catenin signaling activator LiCl partially reversed the effects of Momordin Ic on HaCaT phenotypes and the Wnt/*β*-catenin pathway factors.

The aberrant capacity of keratinocytes to proliferate and to differentiate is the main pathological mechanism of psoriasis [8]. The excessive proliferation of keratinocytes and its regulation mechanism has been the main entry point for the study of psoriasis for a long time, and it is also an important mechanism involved in the treatment of psoriasis by TCM. As we have mentioned, *Kochia scoparia* exerts a certain therapeutic effect on inflammatory skin diseases [11], and the main saponins from *Kochia scoparia*, Momordin Ic, also have anti-inflammatory effects [15]; however, the role of Momordin Ic in the keratinocyte hyperproliferation has not yet been fully determined. Herein, the study examined Momordin Ic cellular functions on HaCaT cells and, for the first time, found that Momordin Ic treatment inhibited HaCaT cell proliferation and promoted cell apoptosis. Notably, Momordin Ic treatment caused G1-phase arrest of the cell cycle, indicating that Momordin Ic might have a potent role in psoriasis treatment, possibly through affecting HaCaT cell proliferation.

Speaking of the mechanism by which Momordin Ic exerts the inhibitory effects on HaCaT cell proliferation, signaling pathways deregulated during psoriasis might be involved. Since Momordin Ic treatment-induced G1-phase arrest of cell cycle, the present study speculated that the Wnt/*β*-catenin pathway was involved. Although *β*-catenin is not necessary for the proliferation of keratinocytes, we found that nuclear *β*-catenin was abnormally increased within the psoriasis epidermis [31]. Dickkopf-1, an inhibitor of Wnt signaling, was significantly reduced within psoriasis lesions [32]. Shen et al. [29, 30] have regarded the Wnt/*β*-catenin pathway as a target for inhibiting HaCaT hyperproliferation and improving psoriasis. Consistent with previous findings, Momordin Ic treatment indeed changed the Wnt/*β*-catenin pathway, significantly downregulating *β*-catenin, c-Myc, and VEGF mRNA and protein expression. More importantly, IF staining showed that Momordin Ic treatment reduced the *β*-catenin levels. Momordin Ic treatment-induced alterations in these factors suggest that the Wnt/*β*-catenin pathway might contribute to Momordin Ic cellular functions.

To further validate that the Wnt/*β*-catenin pathway was involved in Momordin Ic cellular functions, next, we evaluated the dynamic effects of Momordin Ic and the agonist of the Wnt/*β*-catenin pathway. LiCl, an agonist of the Wnt/*β*-catenin signaling, could repress the activity of GSK3*β* and subsequently induce effective stabilization of free cytosolic *β*-catenin [29]. Once inside the nucleus, *β*-catenin binds to TCF/LEF (T-cell factor/lymphoid enhancer-binding factor) proteins, thereby stimulating the expression of Wnt target genes, including cyclinD1 and c-myc [33–35]. Here, under cotreatment of Momordin Ic, LiCl treatment indeed stabilized cytoplasm *β*-catenin, increased *β*-catenin, c-myc, and VEGF proteins, promoted HaCaT cell proliferation, and inhibited cell apoptosis. The cellular and molecular functions of Momordin Ic were all partially reversed by LiCl, suggesting the involvement of the Wnt/*β*-catenin pathway in Momordin Ic functions on HaCaT cells.

Altogether, we demonstrate the inhibitory effects of Momordin Ic, one of the major saponin constituents of Fructus Kochiae, on HaCaT cell proliferation and Momordin Ic-induced alteration within the Wnt/*β*-catenin pathway. Momordin Ic might act on HaCaT cells by modulating the Wnt/*β*-catenin pathway.

## Figures and Tables

**Table 1:** Primers used for qRT-PCR.

Primer	Sequence (5'-3')
*β*-Catenin-F	AGCTTCCAGACACGCTATCAT
*β*-Catenin-R	CGGTACAACGAGCTGTTTCTAC
c-myc-F	GGCTCCTGGCAAAAGGTCA
c-myc-R	CTGCGTAGTTGTGCTGATGT
VEGFA-F	AGGGCAGAATCATCACGAAGT
VEGFA-R	AGGGTCTCGATTGGATGGCA
GAPDH-F	ACAGCCTCAAGATCATCAGC
GAPDH-R	GGTCATGAGTCCTTCCACGAT

## Data Availability

All the available data are included in this study.
